# Role and Mechanism of Olfactory Stem Cells in the Treatment of Olfactory Disorders

**DOI:** 10.1155/sci/6631857

**Published:** 2025-04-24

**Authors:** Shengqi Gan, Siyuan Qu, Hai Zhu, Mengdan Gong, Yizhen Xiang, Dong Ye

**Affiliations:** Department of Otorhinolaryngology-Head and Neck Surgery, The Affiliated Lihuili Hospital, Ningbo University, Ningbo 315040, Zhejiang, China

**Keywords:** olfactory bulb, olfactory disorders, olfactory epithelial, olfactory nerve, olfactory stem cell

## Abstract

Olfactory dysfunction is one of the most prevalent diseases in otorhinolaryngology, particularly since the coronavirus 2019 (COVID-19) pandemic, with a potential impact on daily life. Several etiological factors can contribute to olfactory dysfunction owing to the complexity and specificity of the olfactory transmission pathway. However, current treatments for olfactory dysfunction are limited and their efficacy is unsatisfactory. Olfactory stem cells are multifunctional stem cells in the olfactory mucosa that comprise both horizontal and global basal stem cells (HBCs and GBCs, respectively). These cells can differentiate into various cell types in response to different stimuli with distinct characteristics. The aim of the study was to discuss the mechanisms and functions of stem cells and their application in the treatment of olfactory dysfunction.

## 1. Introduction

This review provides a comprehensive overview of olfactory dysfunction, including its causes, prevalence, symptoms, and clinical treatments. Additionally, it delves into the unique characteristics and regenerative potential of olfactory stem cells and discusses the signaling pathways and regulatory factors involved in their regeneration. Olfactory stem cells have emerged as a promising cellular model for studying neurodegenerative diseases, such as Alzheimer's disease and Parkinson's disease, facilitating early diagnosis and potential treatment for these conditions [1]. Additionally, olfactory stem cells bring new hope for nerve regeneration in patients with spinal cord injuries, as their potential to promote nerve cell regeneration and functional recovery continues to be demonstrated [2]. Therefore, personalized treatment approaches using olfactory stem cells have been proposed.

## 2. Background

Olfactory dysfunction, including hyposmia and anosmia, can be caused by various peripheral and central factors such as viral infections and traumatic brain injuries. The olfactory system is closely connected to the limbic system of the brain and plays a crucial role in odor perception, as well as emotional and social behaviors. Consequently, olfactory dysfunction can substantially impair one's quality of life. However, treatment options remain limited owing to insufficient understanding of the underlying pathological mechanisms.

Traditional treatment methods are the primary means of treating olfactory dysfunction. Nasal corticosteroids, a type of drug therapy, can ease nasal inflammation and congestion, offering short-term relief to some patients [3]. However, they have limited ability to regenerate damaged olfactory neurons. Olfactory training, a form of behavioral treatment, stimulates the olfactory system [4]. While it has an impact, the results appear slow and less effective in severe cases [5]. Surgical approaches, such as nasal surgery for obstructions, can be effective in certain scenarios [6]. Nevertheless, they do not facilitate nerve regeneration and carry surgical risks [7].

Recent advancements in stem cell biology have opened new avenues for therapeutic interventions. Olfactory stem cells located in the olfactory epithelium possess remarkable self-repair and self-renewal capabilities that are essential for restoring olfactory function [8]. Numerous studies have explored the signaling pathways and regulatory mechanisms of olfactory stem cells, aiming to use them in the treatment of neurodegenerative diseases and spinal cord injuries [1]. Thus, olfactory stem cells hold great promise for the treatment of olfactory dysfunction.

Moreover, gene therapy has achieved certain research results in animal models. It is highly targeted, aiming to replace diseased genes and stimulate the regeneration and recovery of the olfactory system [9]. Platelet-rich plasma (PRP), as an autologous blood product, is safe and noninvasive in clinical practice [10]. The growth factors it is rich in can stimulate the repair of olfactory epithelial stem cells and nerves, demonstrating great potential [11]. Currently, biomaterials are used to support olfactory stem cells in the treatment of olfactory disorders, but challenges such as material selection and compatibility still remain [12].

Thus, emerging therapies, particularly those harnessing the regenerative potential of olfactory stem cells, present innovative approaches for personalized treatment, targeting the underlying causes of olfactory dysfunction. However, the integration of these therapies into clinical practice faces several challenges, including ethical concerns, high costs, and issues related to clinical feasibility (Table 1).

This article focuses on olfactory stem cells, examines their roles and mechanisms in olfactory disorders, summarizes recent research findings, and analyzes the feasibility and challenges of using olfactory stem cells for therapeutic purposes.

## 3. Epidemiology

Olfactory disorders are highly prevalent in the global populations. In 2017, Huang et al. [13] surveyed 101,510 Chinese adults and found that 2.4% (*n* = 302) had olfactory disorders. According to the 2013 United States (US) Census Bureau population estimates, ~1.4% (~4.4 million) of adults in the US have varying degrees of olfactory impairment [14]. According to a sample survey conducted in Sweden in 2004, ~5%–20% of the general population exhibited varying degrees of olfactory sensory deprivation, and ~2.5%–5% of the population exhibited a complete loss of their olfactory sense [15].

The factors leading to olfactory dysfunction vary and show different clinical manifestations (Table 2).

Upper respiratory tract infections such as viral colds can cause olfactory dysfunction. During the coronavirus 2019 (COVID-19) pandemic, the incidence of olfactory dysfunction has increased sharply. In 2020, after reviewing and analyzing 3,563 patients with COVID-19, researchers found that the prevalence of olfactory dysfunction was 47%, among which 67% have mild to moderate symptoms, and 31% have severe symptoms [22]. However, they used a self-reported patient approach, which undermined the true rate of olfactory dysfunction.

Two studies using different objective assessments reported that the percentage of COVID-19 patients with olfactory impairment was 73% and 98% respectively [23, 24]. The discrepancy in these figures may be due to the studies being conducted in Italy and Iran, and the use of different olfactory tests: the Connecticut Chemosensory Clinical Research Center oronasal olfaction test (CCCRC) and the University of Pennsylvania Smell Identification Test (UPSIT). However, both studies found that the positive rate of olfactory dysfunction detected through objective assessment methods was significantly higher than the rate observed in self-reported questionnaires [25]. Moein et al. found that only 35% of 60 COVID-19 patients reported a loss of smell; however, 99% of the patients had different degrees of olfactory disorders. In a multicenter European study, the researchers also found that 85.6% of 417 patients with mild to moderate COVID-19 had olfactory dysfunction, and 11.8% of them had olfactory dysfunction as the first symptom. Jane et al. also concluded that considering olfactory dysfunction as an early symptom in patients with COVID-19 could facilitate in early clinical diagnosis and treatment. After analyzing 10 studies concerning olfactory dysfunction, they found that 52.73% of the patients had COVID-19 [17].

Several studies have found that chronic rhinosinusitis with nasal polyps (CRSwNP) is associated with a higher incidence and severity of olfactory dysfunction than chronic rhinosinusitis without nasal polyps (CRSsNP), which may be the result of multiple pathophysiological factors [26]. A major reason for this finding may be that the mechanical obstruction of the neuroepithelium of the olfactory fissure resulting from mucus, edema, and nasal polyps is more severe in patients with CRSwNP [19]. Computed tomography (CT) has also shown that in patients with CRSsNP, the olfactory fissure is less turbid, and there is less obstruction of odor transmission [27].

Aging, neurodegenerative diseases, and smoking are important etiological factors for olfactory dysfunction, all of which increase the risk of developing olfactory dysfunction. According to a survey, the prevalence of olfactory dysfunction in the general population is 3.8%–5.8% [28] with a high prevalence rate of 13.9% among people over 65 years of age, over 50% among people aged 65–80 years, and 80% among people over 80 years of age [16]. Numerous studies have also demonstrated that olfactory dysfunction is an early and common indicator of many neurodegenerative diseases, including Alzheimer's disease [29] and Parkinson's disease [20]. Gaurav et al. observed that compared with nonsmokers (odds ratio [OR] 1.59, 95% confidence interval [CI] 1.37–1.85), smokers were 59% more likely to experience olfactory dysfunction [30].

Substantial differences between the sexes have been reported in the etiology of olfactory disorders. The prevalence of olfactory disorders is higher in male than in female. Howard et al. (2012) found that 12.4% of their population had olfactory dysfunction (55% male, 45% female) [31]. In males, olfactory dysfunction is most often caused by inflammation and toxic exposure, whereas in females it is more often caused by viral infections [32]. In addition, females reported higher olfactory sensitivity than males [33].

Patients with head tumors can also experience olfactory dysfunction, as evidenced by a study that demonstrated that dysfunction can be detected in more than 90% of patients with head tumors [18].

Finally, environmental quality is closely related to olfactory dysfunction. Exposure to polluted environments, including air pollutants such as PM2.5 and sulfur dioxide, may lead to olfactory dysfunction [34], whereas long-term exposure to toxic substances such as barbiturates can result in irreversible loss of smell [21].

## 4. Adverse Events Related to Olfactory Dysfunction

Olfactory disorders may seem less apparent than loss of sight, taste, and/or hearing, but the importance of olfactory sensations should not be downplayed. Olfactory processes are directly related to appetite, emotions, and personal safety.

Zang et al. [35] found that olfactory dysfunction can affect food perception and lead to changes in eating habits. Olfactory experiences during the neonatal period may influence the development of future cognitive networks in newborns [36]. Additionally, the researchers observed a correlation between higher scores on the Olfactory Disorders Questionnaire and an increased risk of depression [37]. Among patients with olfactory disorders caused by COVID-19, the prevalence of depression is as high as 43%. Additionally, 57% of these patients were unable to detect harmful gases, such as smoke from fires and leaking natural gas, which further elevates their risk to personal safety [38].

## 5. The Necessity for Research

Current clinical treatments for olfactory disorders and their efficacy are limited. Primarily, topical corticosteroids, surgery, and olfactory training are the available treatments. Other emerging therapies, such as *N*-acetylcysteine and PRP, have revealed some progress; however, further research is needed to confirm their therapeutic effects [39].

However, olfactory stem cells have great potential, and as the most easily accessible neural stem cells in the human body [40], they play an important role in olfactory maintenance. Stem cell transplantation has been used with some progress in treating neurological disorders [41], providing new ideas for treating olfactory disorders.

## 6. Introduction to Olfactory Stem Cells

Stem cells are undifferentiated cells that possess the characteristics of self-renewal, self-cloning, and differentiation, and exist at all stages of human life [42]. Olfactory stem cells, including olfactory mucosal mesenchymal stem cells (OM-MSCs), olfactory ensheathing cells (OEC), and olfactory sensory neural stem cells, differ in their origins characteristics, and functions [43].

OM-MSCs can be isolated from the lamina propria of the nasal mucosa using *in vitro* methods [43]. They demonstrate robust proliferation and differentiation potential and can be induced to differentiate into adipocytes, osteoblasts, and neurons, among other cell types, under *in vitro* conditions. Consequently, OM-MSCs are regarded as optimal seed cells for tissue repair. Additionally, Nivet et al. [44] identified that OM-MSCs also induce neurogenesis through a trophic effect that contributes to hippocampal nerve repair.

OECs originate from the olfactory placodes [45]. Numerous studies have demonstrated that OECs facilitate nerve repair by secreting bioactive factors and modulating inflammatory and immune responses [46].

Olfactory sensory neural stem cells (i.e., HBCs and GBCs), the cell subpopulations that are the focus of this study, are usually cultured and expanded *in vitro* through a noninvasive method of harvesting olfactory mucosal tissue from the olfactory epithelium within the nasal cavity [47].

The olfactory epithelium is distributed on the inner side of the upper turbinate, lower surface of the cribriform plate, upper part of the nasal septum, and some inner sides of the middle turbinate. The epithelium comprises four cell types: (1) olfactory neurons, (2) supporting cells, (3) microvilli cells, and (4) olfactory epithelial stem cells.

Olfactory epithelial stem cells, also known as olfactory stem cells, are composed of both horizontal and global basal cells (HBCs and GBCs, respectively) [48] and are known as olfactory stem cells. These cells continuously differentiate into new olfactory neurons and other olfactory epithelial cells that cope with the degradation and loss of olfactory function caused by various external stimuli, thereby maintaining the stability of olfactory function. HBCs are located immediately above the basement membrane, and GBCs are located above the HBCs. A study found that the regeneration of olfactory receptor neurons (ORNs) is usually carried out by GBCs. Exogenous transforming growth factor-B (TGF-B) and platelet-derived growth factor (PDGF) induce the transformation of GBCs into neurons through programmed regulation; when the olfactory epithelium is severely damaged, the HBCs become activated and proliferate, after which they ultimately differentiate into various cellular components of the olfactory epithelium [47]. Leung et al. [47] identified a pathway through which HBCs expressing keratin K5, differentiated into GBCs by combining HBC-specific CreER spectral tracing with transgenic markers for GBCs.

## 7. Types of Olfactory Dysfunction

Olfactory dysfunction can be classified into qualitative and quantitative disorders based on their ability to perceive and identify odors. Qualitative dysfunction includes parosmia and phantosmia, Whereas, quantitative dysfunction includes hyposmia, functional anosmia, anosmia, and hyperosmia [49].

According to its etiology and anatomical location, olfactory dysfunction is classified into peripheral and central causes [50].

Peripheral factors refer to lesions occurring in olfactory receptors and their supporting structures, including the olfactory epithelium (OE) and nerves. These lesions can be categorized into several types: (1) Obstructive factors: [19] Olfactory dysfunction resulting from physical obstructions, such as a deviated nasal septum or nasal polyps. (2) Inflammatory lesions: Conditions such as chronic rhinosinusitis (CRS) [51] can lead to olfactory dysfunction, often accompanied by symptoms such as mucosal edema, thickening, and increased secretions. (3) Infectious factors: A considerable category of peripheral causes include loss of smell following upper respiratory infections [17] which may be related to transient edema of the olfactory nerve and inflammation-induced conduction block. (4) Chemical substance effects: [21] The impact of chemical agents on olfaction primarily occurs through alterations in the normal physiological state of the olfactory mucosa, resulting in receptor dysfunction.

Central causes involve central processing components of the olfactory system, including the olfactory bulb (OB), olfactory tract, and associated brain structures (such as the olfactory cortex and limbic system). The etiological factors generally include the following: (1) Neurodegenerative diseases: [20] Conditions such as Parkinson's disease often lead to neuronal damage and death, resulting in olfactory disorders. (2) Head trauma: [52] Traumatic injuries may directly damage the olfactory bulb or associated neural pathways, and subsequent inflammation and swelling can further impair the transmission of olfactory signals. (3) Cerebrovascular events: [53] Events such as stroke may affect the brain regions responsible for processing olfactory information, leading to olfactory dysfunction. In particular, ischemic stroke can result in neuronal hypoxia, damaging the olfactory processing areas. (4) Tumors: [18] The presence and growth of tumors may compress or interfere with the olfactory nerves or adjacent regions. (5) Central nervous system Infections: [54] Infections such as meningitis or encephalitis may damage olfactory neurons through inflammatory responses, thereby affecting the transmission of olfactory signals [22]. (6) Specific neurological diseases: Certain conditions, such as multiple sclerosis [55], may also lead to olfactory dysfunction owing to potential demyelination, which affects the transmission of neural signals.

In addition, the Chinese expert consensus adds another category to this classification, namely mixed olfactory dysfunction, which includes two or more of the three categories of olfactory disorders listed above [56].

## 8. Olfactory Transmission Pathway

The olfactory system consists of three structures [56]: (1) the olfactory epithelium, (2) the olfactory bulb, and (3) the higher olfactory structures, including the anterior olfactory nucleus and pyriform cortex [57].

The olfactory cleft is situated between the superior nasal concha's superior medial vertical plate, internal surface of the superior nasal concha, internal surface of the middle nasal concha, and corresponding nasal septum. The mucosa in this region contains the olfactory epithelium (OE) and lamina propria (LP). The OE is a type of neuroepithelium rather than a respiratory epithelium [58]. Odorant compounds are transported into the nasal cavity via the airflow. These compounds are then detected by olfactory neurons in the olfactory cleft region, which they bind to receptors on the cilia of olfactory neurons and generate action potentials in the axons, which transmit signals to the olfactory bulb of the brain, resulting in a sense of smell. Olfactory neurons are bipolar receptor cells whose axons cross the cribriform plate to form the olfactory tract, which then form synapses with other neurons in the olfactory bulb to form the olfactory nerve (cranial nerve I) [59].

**Figure 1:**
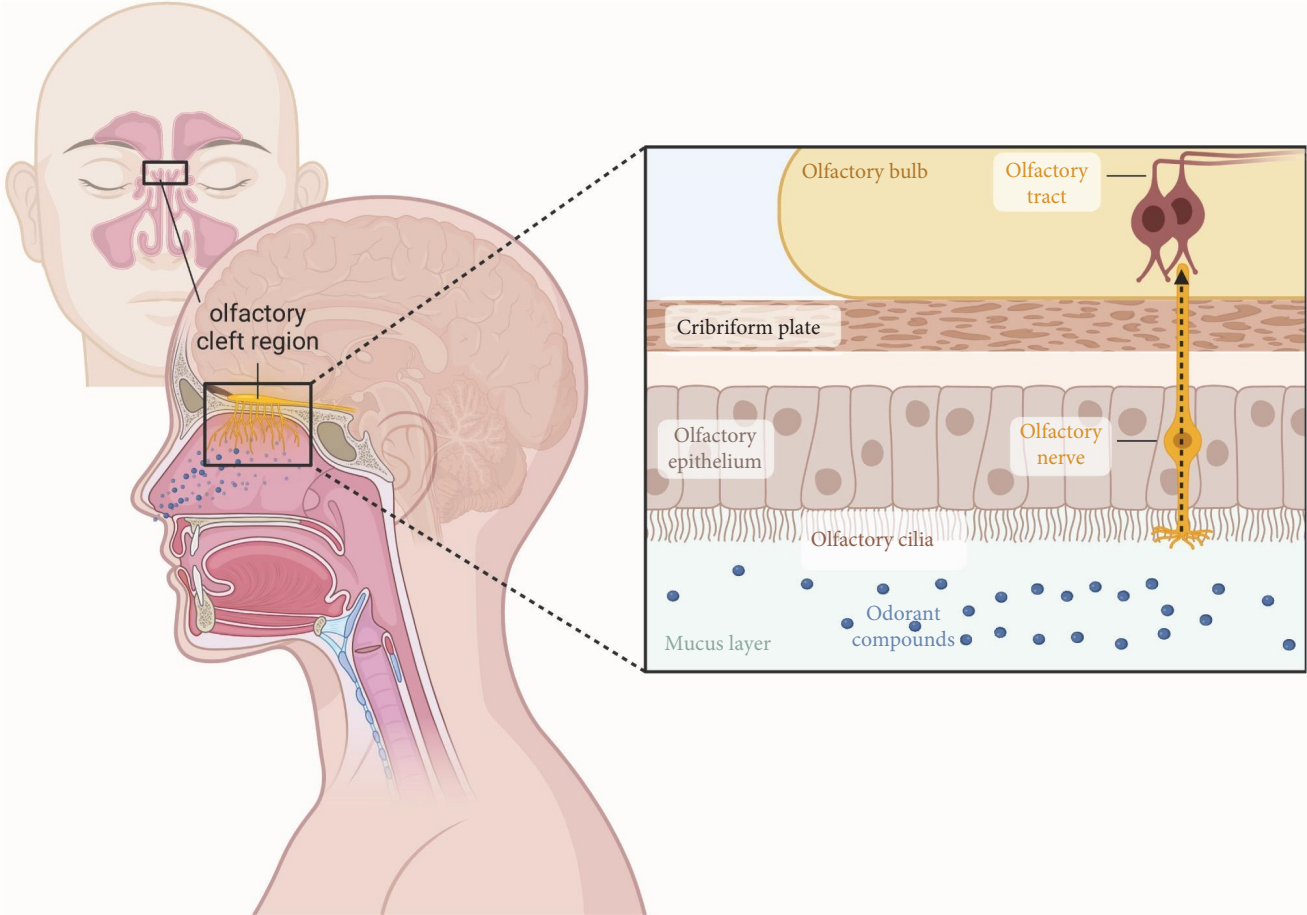
Localization of the olfactory cleft region and nasal olfactory transmission pathways.

Subsequently, the olfactory bulb receives signals and conducts signal transduction and encoding (Figure 1) [60] first transmitting information to the secondary olfactory structures, including the superior olfactory nucleus, piriform core olfactory tube, lateral endogenous core, and para–amygdaloid complex. These secondary olfactory structures are then transmitted to the tertiary olfactory structures, including the orbitofrontal core, internal core, and dorsal hippocampus [61].

## 9. Potential and Characteristics of Olfactory Stem Cells

The OE has a strong lifelong regenerative capacity, and after injury, olfactory epithelial stem cells are capable of self-renewal and directed differentiation, allowing the olfactory nerve to recover both anatomically and functionally [62]. The mammalian olfactory neurons have a lifespan of 30–90 days, and newborn neurons differentiated from basal cells replace senescent dead neurons, thus maintaining homeostasis within the OE [63]. Murrell et al. induced olfactory epithelial stem cells to differentiate into skeletal muscle and myocardial cells, which also demonstrated the differentiation potential of olfactory stem cells and expressed the corresponding antigens and biological activities [64]. Among them, nerve growth factor, brain-derived neurotrophic factor (BDNF), and neurotrophic factor-3 (NT-3) are involved in regulating the apoptosis of mature ORNs and increasing the proliferation of precursor cells, whereas growth and differentiation factor II (GDFII) inhibits the transformation of neural precursor cells into neural cells [65].

Olfactory epithelial neural stem cells can be obtained from the nasal cavity using a noninvasive sampling method. Katarina et al. stripped the olfactory nerve epithelium from the nasal septum and successfully cultured it *in vitro* [66]. Mumm et al. [67] used a serum-free culture method to develop neural clone-forming cells from the OE of embryonic mice, which eventually differentiated into neurons. Richard et al. also demonstrated that sphere formation in the olfactory bulb is a marker for olfactory progenitor cells to colonize and differentiate multidirectional *in vitro* and is required for their terminal transformation [68].

Using the leucine-rich repeat-containing G-protein coupled receptor 5 (Lgr5) as a marker for adult stem cells [69], Chen et al. [70] found that Lgr5+ cells have the characteristics of cyclic stem cells and thus maintain the regeneration of many cell types during normal physiological cycles or after olfactory dysfunction. Furthermore, Wnt signaling was shown to promote the proliferation of Lgr5-positive GBCs and subsequent olfactory epithelial neurogenesis [71].

## 10. Recent Applied Uses of Olfactory Stem Cells in the Therapy of Olfactory Dysfunction

Olfactory stem cells (OSCs) exhibit considerable therapeutic potential for treating peripheral factor-induced olfactory disorders. Several studies have been conducted on the regenerative properties of olfactory stem cells (Table 3). HBCs are considered the stem cells of adult OE [81]. Some authors have named these cells as the stem cells of the epithelium, although this naming is based on limited evidence [82]. HBCs indeed play a crucial role in olfactory regeneration. Carter et al. [83] cultured various cell types, including neurons, from HBCs derived from the neonatal epithelium.

Sarah Kurtenbach et al. successfully improved olfactory function in a mouse model of olfactory disorders caused by ciliary motility gene deficiency (i.e., peripheral factors) through transplantation of purified GBCs. Cell-based therapy restored olfactory function in an inducible model of hyposmia [84]. In clinical applications, olfactory stem cells have also been proven to be safe. A 3-year clinical trial showed that in paraplegic patients who received autologous olfactory ensheathing cell (OEC) transplantation, no tumors or complications were found in imaging examinations, which demonstrated its safety [85].

However, the transformation from basic research to clinical application still faces many challenges. The survival rate after stem cell transplantation is a crucial factor affecting the therapeutic efficacy. In animal models, the survival rate of OECs is as low as 2.5% 4 weeks after transplantation [86]. Therefore, it is crucial to modulate the cellular microenvironment, such as oxygen levels and signaling cues, to enhance cell survival [87].

Moreover, immune rejection remains a major obstacle in stem cell transplantation. Frederiksen's latest research shows that even with the knockout of B2M and CIITA genes (encoding major histocompatibility complexes I and II) to avoid an immune response, T-cell infiltration occurs on day 8 and immune rejection on day 11 [88]. Meanwhile, exosomes derived from olfactory stem cells have low immunogenicity and immunomodulatory functions. Combining them with stem cells can better repair and nourish nerves to treat retinal diseases, offering a new solution to stem cell transplantation-related immune risks [89].

However, the application of stem cells in olfactory disorders caused by central factors may be relatively limited. For example, gamma-aminobutyric acid (GABA) selectively influences axonal growth [90]. Catherine et al. investigated neuronal growth in ORNs in the presence of GABA receptor agonists and antagonists and found that the release of GABA-inhibited axonal growth in ORNs [91]. Therefore, certain neurotransmitters and signaling pathways within the central nervous system may inhibit the differentiation of olfactory stem cells or axonal growth. However, James et al. found that the *Δ*Np63 isoform of the transcription factor p63 acts as a primary regulator of HBC activation. Its elimination triggers the pluripotency of HBCs [92]. Thus, *Δ*Np63 emerges as a potential therapeutic target for reversing neurogenic olfactory loss in the elderly OE [93]. Neurogenin 1 (Ngn1) and NeuroD1 promote the differentiation of olfactory progenitor cells to their terminal stages, whereas the transcriptional repressors Hes1 and Hes5 inhibit neuronal differentiation and promote progenitor cell proliferation [94]. This suggests that exogenous stem cells intervene in central olfactory disorders by modulating the neurogenic microenvironment and promoting endogenous neural regeneration [95].

In addition, the interaction between stem cells and residual HBCs presents a potential avenue for the treatment of olfactory dysfunction. Intravenous injection of adipose-derived stem cells can induce the differentiation of olfactory sensory neurons (OSNs) and facilitate the colonization of the olfactory neuroepithelium in damaged areas, thereby addressing olfactory functional impairment [96]. Furthermore, the use of nanomaterials in piggyback olfactory stem cells has shown promise for enhancing cell survival. Chitosan not only promotes the growth of neurons and Schwann cells [97], but may also guide axonal growth. Soluble chitosan supports the differentiation of olfactory stem cells, synaptic growth, and signaling, thereby facilitating the maturation of olfactory neuronal precursor cells (ONCs) into functional olfactory receptor neurons (ORNs) and), offering new options for the treatment of olfactory disorders [98].

## 11. Potential Mechanisms of Olfactory Stem Cell in Olfactory Dysfunction

**Figure 2:**
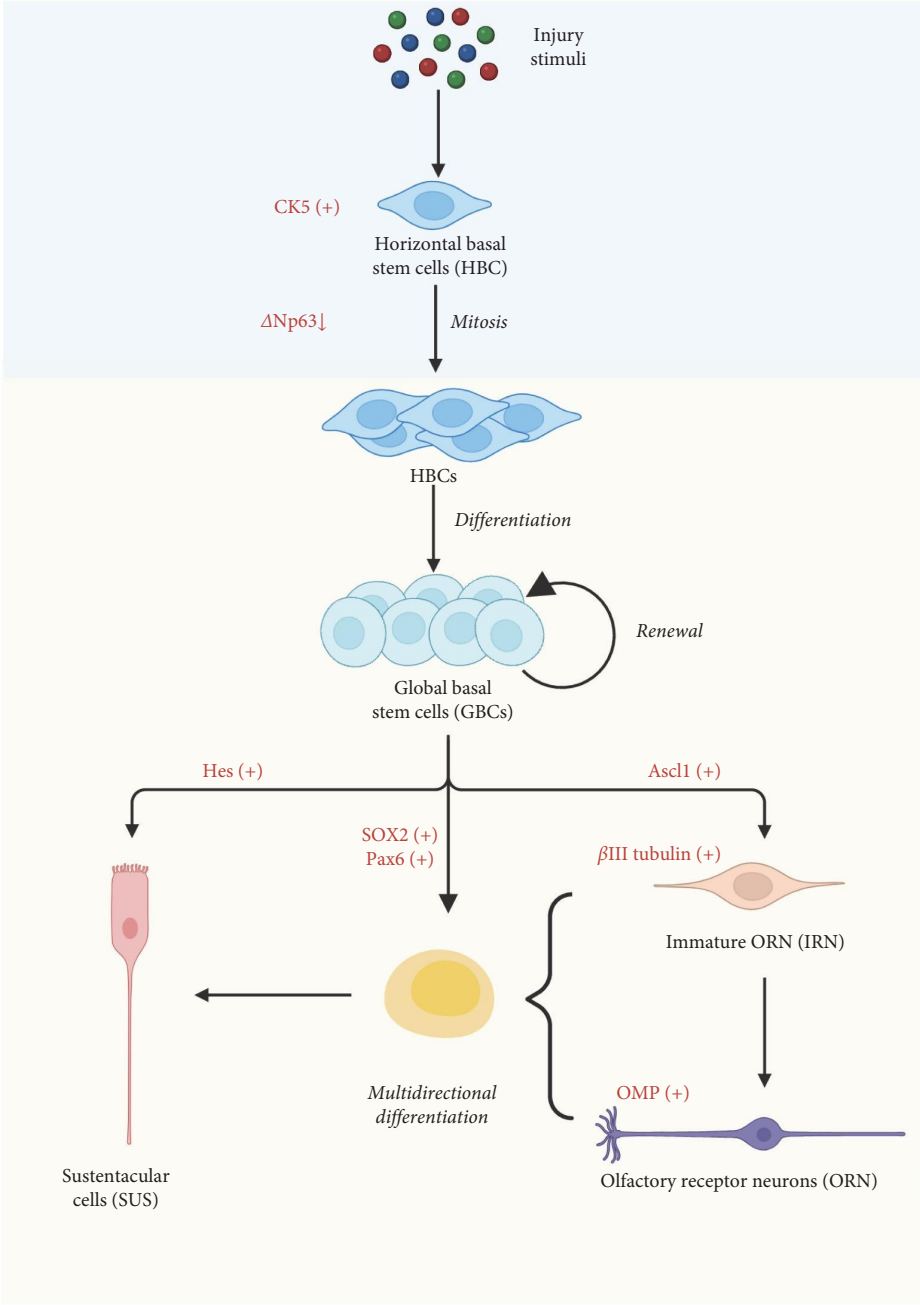
Differentiation of both HBCs and GBCs after injury. *Δ*Np63, tumor protein p63 with exon replacement at the *Δ*N-terminus; Ascl1, achaete scute homolog−1; CK5, cytokeratin 5; Hes, basic helix–loop–helix (bHLH) transcriptional repressors; IRN, immature ORN; OMP, olfactory marker protein; ORN, olfactory receptor neuron; Pax6, paired box protein 6; SOX2, SRY-related HMG-box gene 2.

As long as the olfactory epithelium is stimulated, the expression of *Δ*Np63 decreases, leading to the proliferation of HBCs during division, with notable expression of keratin 5 (CK5), marking them as progenitor cells of the olfactory neuroepithelium. Subsequently, these progenitor cells differentiate into GBCs. GBCs have the potential for multidirectional differentiation. When GBCs express Ascl1, they first differentiate into immature olfactory receptor neurons (IRNs), at which point *β*III tubulin is highly expressed as a marker of IRNs [99]. Subsequently, IRNs develop into mature olfactory receptor neurons (ORNs), characterized by high expression of olfactory marker protein (OMP) [100]. Neuron generation is accelerated through paired box protein 6 (Pax6), and the neuronal progenitor pool is expanded through SOX2 [101]. Moreover, the expression of Hes1 [102], a downstream transcription factor of Notch1 and 2, and the Notch pathway promote the differentiation of GBCs into sustentacular cells (SUS), as shown in Figure 2 [103].

When acute inflammation occurs after injury, regeneration signals are activated partially through tumor necrosis alpha (TNF-α). Notably, the tumor necrosis factor receptor 1/nuclear factor kappa beta (TNFR1/NF-κß) pathway plays a crucial role in inducing interleukin-6 (IL-6) expression, which promotes nerve regeneration [104]. It facilitates the proliferation and differentiation of HBCs into GBCs. This sequential process enables the replacement of olfactory receptor neurons (ORNs) in response to environmental cues, thereby preserving the homeostasis of the olfactory neuroepithelium [105]. Olfactory mucus secreted by Bowman's glands protects cilia and transports odor molecules [106]. At the same time, eosinophils are activated and begin to proliferate [107].

In addition to TNF-α signaling, the Wnt and Notch signaling pathways also play pivotal roles in regulating the regeneration of OSNs [108]. Wnt ligands bind to Frizzled receptors on cell surfaces, recruiting LRP5/6 coreceptors and activating dishevelled. This inhibits the GSK-3*β* complex, preventing β-catenin phosphorylation and degradation [109]. β-Catenin then accumulates in the cytoplasm, translocates to the nucleus, where it accumulates and upregulates the expression of Cyclin D1 [110]. This process promotes the elongation and convergence of OSN axons [111].

**Figure 3:**
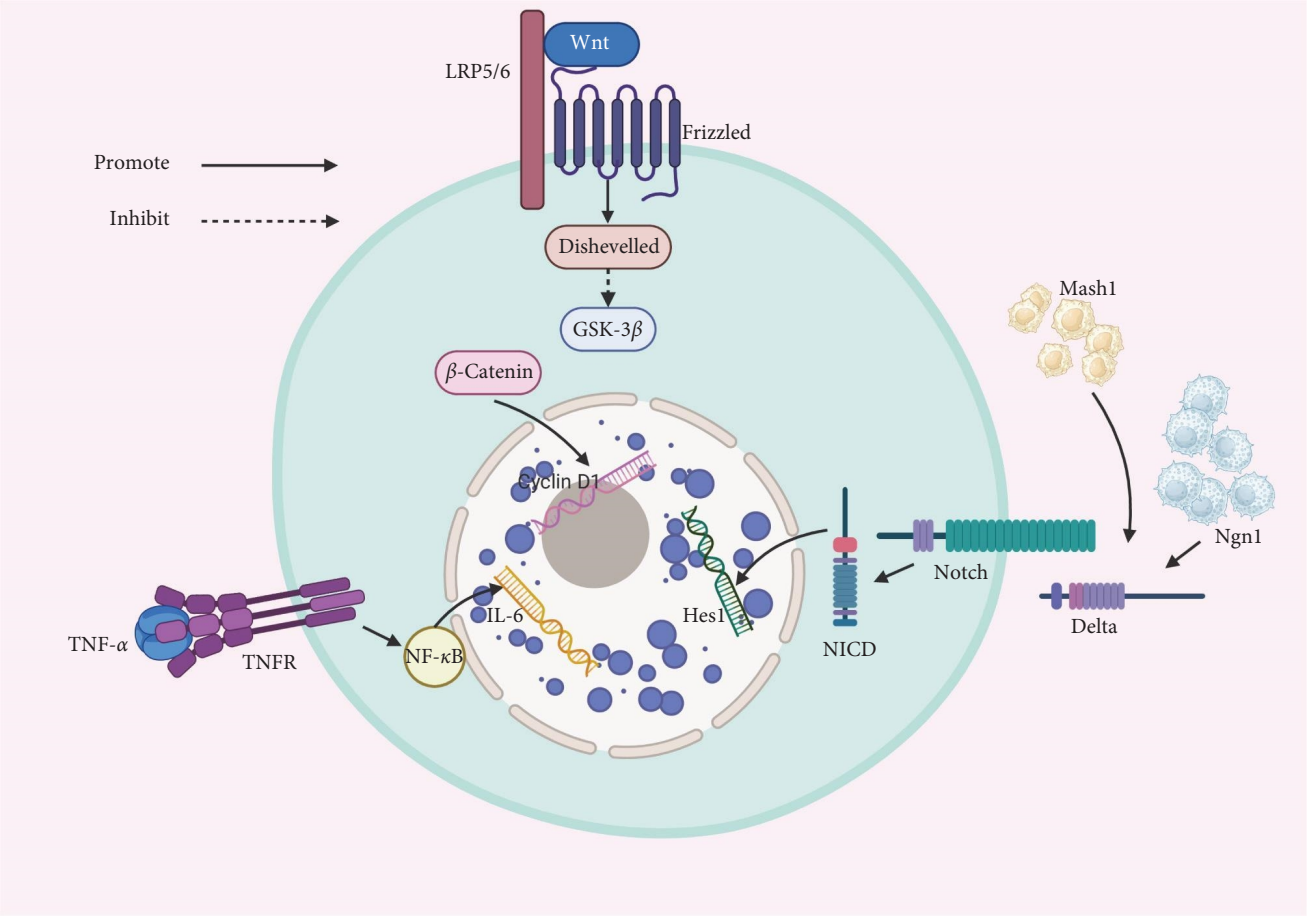
Signal transduction of olfactory stem cells in olfactory dysfunction. *β*–catenin, beta–catenin; Delta, Delta-like protein; GSK3*β*, glycogen synthase kinase 3 beta; Hes1, basic helix–loop–helix (bHLH) transcriptional repressors 1; IL-6, inducing interleukin-6; LRP5/6, low-density lipoprotein receptor–related protein; Mash 1, mammalian achaete scute homolog 1; Ngn1, Neurogenin 1; NICD, notch intracellular domain; TNF-*α*, tumor necrosis alpha; TNFR1/NF-*κ*ß, tumor necrosis factor receptor 1/nuclear factor kappa beta.

Furthermore, since Notch pathways activated by upstream Neurogenin 1 (Ngn1) and mammalian achaete scute homolog 1 (Mash 1) [112], they function as GBCs during OE growth and as HBCs in OE repair after injury [113]. When the Notch receptor on a cell binds to its ligand Delta on an adjacent cell, proteases cleave the receptor, releasing Notch intracellular domain (NICD) [114, 115]. NICD then enters the nucleus, where it activates Hes1, a key regulator of neurogenesis and differentiation. Thus, Notch signaling determines the balance between neurons and nonneuronal cells during restoration of the epithelium after injury [116] (Figure 3).

However, when aging or chronic inflammation occurs [73], HBCs are altered such that neuronal activity is reduced and respiratory-like chemotaxis occurs, a process that gives rise to ciliated respiratory and secretory cells rather than to ORNs. Allison et al. found that aging GBCs probably continue to function as transient neuronal precursor cells based on their transcriptional profiles, whereas HBCs are dormant, both of which lead to the failure of the olfactory neuroepithelium to maintain homeostasis, thus causing olfactory dysfunction [74]. Thus, OE stem cells may serve as a therapeutic target for olfactory restoration.

**Figure 4:**
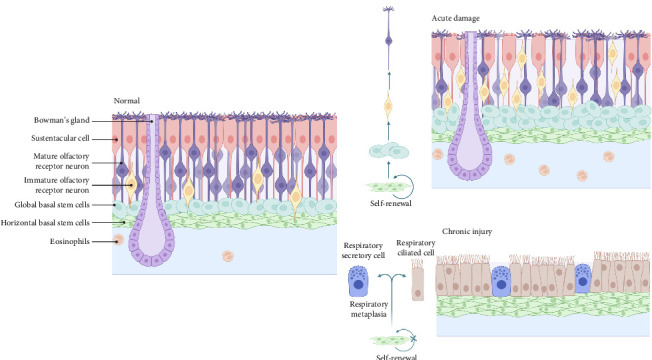
The normal morphology of olfactory epithelium and its changes under acute damage and chronic injury.

Biopsy results from the olfactory mucosa of patients with chronic rhinosinusitis (CRS) and aging patients indicate a substantial decrease in olfactory receptor cells, which is seen as an indication of damage to the olfactory epithelial mucosa [117]. This process may have resulted from prolonged exposure to chronic cytokines, which resulted in the initial death of olfactory epithelial cells, followed by inhibition of olfactory stem cell regeneration and metaplasia into the respiratory epithelium, such as respiratory ciliated and secretory cells [75] (Figure 4). Kevin et al. also found that long-term neuronal turnover in aging models causes atrophy of the glomerulus, which impairs dopaminergic neuronal function in the periphery; thus, OE regeneration is diminished after the disappearance of GBCs [72].

## 12. The Potential Risks of Olfactory Stem Cells

Assessing Tumorigenicity in Stem Cell-Derived Therapeutic Products: A Critical Step in Safeguarding Regenerative Medicine

First, the high proliferative capacity and pluripotency of stem cells may lead to unexpected differentiation and even result in the formation of tumors [118]. For example, a case study found that an 18-year-old female patient developed an intraspinal mass 8 years after receiving olfactory stem cell transplantation [119]. The malignant transformation process may occur at multiple stages: During preparation, donor stem cells may acquire genetic abnormalities. Long-term in vitro expansion can cause karyotypic instability, and proliferating stem cells may differentiate into malignant tumors in response to in vivo microenvironmental signals [120]. Therefore, before clinical application, the culture process and differentiation state of stem cells must be strictly controlled to reduce the incidence of tumors.

Second, the clinical stem cell transplantation therapy involves complex ethical issues. The most fundamental ethical concern is whether the acquisition of embryonic stem cells, involving the destruction of human embryos, can be morally acceptable to the general public [121]. In addition, the mechanisms for patients' informed consent, risk notification, and the protection of the privacy of subjects need to be further improved to ensure the information security of participants and enable them to fully understand the potential risks of the treatment [122].

Long-term safety is undoubtedly a major challenge in stem cell therapy. Currently, the follow-up clinical and animal trials are generally under 5 years, lacking long-term side-effect monitoring. A Chinese research team used MRI to monitor 16 patients who received OEC transplantation. No adverse reactions were detected over 38 months. However, due to the small sample size, the long-term efficacy requires further validation [123].

## 13. Unresolved Issues of Olfactory Stem Cells

Olfactory stem cells offer a new approach for the treatment of olfactory disorders; numerous questions about their safety, feasibility, and efficacy still need to be addressed in more clinical trials: (1) Selection of olfactory stem cell source–should autologous or nonautologous be used and whether they cause immune rejection, how to deal with it; (2) the growth cycle of implanted stem cells remains uncertain, and it is unclear whether multiple treatments are required for optimal effectiveness; (3) properly activating olfactory stem cells and guiding their growth and development is vital for preventing potential tumor growth; (4) further research is needed to determine the exact location, method, and dosage (including the purity, activity, and number of stem cells) for stem cell implantation, as this information is currently unknown; and (5) individualized olfactory stem cell transplantation regimens need to be developed for different etiologies in different patients (e.g., infections, aging, head tumors).

## 14. Conclusions and Future Directions

Stem cell-based treatments for olfactory dysfunction demonstrate substantial potential. Sarah et al. transplanted purified adult mouse olfactory stem cells in the form of nasal drops thereby improving the olfactory function in mice. However, this technique has a low survival rate for transplanted cells, which limits the efficacy of the therapy [84]. Mariyam et al. attempted to improve the survival rate of transplanted cells by pretreating the olfactory ensheathing cells (OECs) under low-oxygen conditions [87]. These methods may also be applicable to olfactory stem cells. Numerous studies have demonstrated that the upregulation of neurotrophic factors [89] and brain-derived neurotrophic factors [90] can facilitate the repair of olfactory dysfunction through stem cell transplantation in the affected area. These neurotrophic factors have been shown to support the survival, growth, and differentiation of transplanted stem cells, thereby enhancing their efficacy.

Alternatively, biomaterials and scaffolds can be used to transport several extracellular matrixes that can survive and differentiate, thereby improving the survival rate and therapeutic effects of stem cell transplantation [12]. For instance, the dynamic mechanical properties of hydrogels can play a crucial role in stem cell fate [124], and the ligand type of three-dimensional (3D) scaffolds primarily determines the differentiation profile of neural stem cells [125]. Therefore, hydrogels and 3D scaffolds can be specifically stimulated to induce cellular differentiation and deliver stem cells to the olfactory system.

Given the unique regenerative capacities of GBCs and HBCs described previously, olfactory stem cell regeneration and differentiation can be promoted through the following approaches: (1) Optimizing the culture medium formulation *in vitro* to provide the most favorable growth conditions for stem cells. (2) Modulation of signaling pathways *in vivo* to regulate the physiological activities of stem cells. (3) Construction of a bioactive microenvironment that supports stem cell growth and development. In adults, the ON retains embryonic capacity to continuously renew olfactory neurons from stem cells located deep within the epithelium [126]. This phenomenon provides a theoretical basis for the regenerative therapy of olfactory neurons, which are the primary active proliferative group within the olfactory epithelium and are capable of self-renewal and differentiation into various cell types, including HBCs. Differentiation trajectories and lineage commitments were tracked using specific molecular markers (Table 4). In contrast, HBCs are largely quiescent under normal conditions and are activated only upon receiving appropriate stimuli. This activation process involves a reduction in the expression of *Δ*Np63, followed by the subsequent activation of Notch1, TNFR1, Wnt, and other signaling pathways.

## Figures and Tables

**Table 1:** Advantages and limitations of current and emerging therapies for olfactory dysfunction.

	Treatment method	Advantages	Limitations
Current treatment methods	Drug treatment [3]	Convenient and easy to implement; effective for certain cases of inflammatory or infectious olfactory dysfunction	Limited effect on neurogenic olfactory dysfunction; The side effects caused by long-term use of drugs
	Olfactory training [4, 5]	Noninvasive and safe	It demands long-term perseverance as the effect takes a slow start
	Surgical treatment [6]	Effective for structural causes (e.g., nasal polyps, sinusitis)	Invasive with surgical risks

Emerging treatment methods	Olfactory stem cell therapy [8]	The potential to repair and regenerate in cases of neurogenic olfactory dysfunction; Possess long-term efficacy	Currently in the research stage; Problems such as immune rejection and ethical concerns exist.
	Gene therapy [9]	It provides a fundamental treatment for genetic olfactory dysfunction; This therapy is highly targeted	Currently in the research stage; Complex technology, high costs
	Biomaterial [12]	Provides a growth environment for stem cells, promotes tissue repair	Material selection and biocompatibility still need optimization

**Table 2:** Factors that contribute to olfactory disorders and reports on related clinical observations.

Factor	Contribution to olfactory disorders	Clinical observations
Aging	Olfactory function declines with aging	Reduced sense of smell and ability to recognize odors, leading to reduced enjoyment and safety of foods [16]

Upper respiratory infections	Damage to olfactory receptors	Temporary or permanent loss of smell, usually accompanied by other symptoms such as nasal congestion, runny nose, cough, and fever [17]

Head trauma	Physical damage to the olfactory nerve	Loss of sense of smell, usually accompanied by other neurologic symptoms such as headaches, visual disturbances, seizures [18]

Sinusitis, nasal polyps	Inflammation of the nasal passages	Nasal congestion and inflammation impede olfactory pathways for odor transmission [19]

Neurodegenerative diseases	Degradation of olfactory pathways	Loss of smell can occur early in diseases such as Alzheimer's and Parkinson's disease [20]

Environmental toxins	Exposure to chemicals and pollutants	Olfactory receptors and the olfactory nerve may be damaged [21]

**Table 3:** Experimental studies related to the regenerative properties of olfactory stem cells.

Research target	Species	Methods	Results	Reference
HBCs	OMP-tTA; TetO-DTA transgenic mice model	Coronal sections of the OE	Aged mice lack mature and immature neurons, as well as GBCs, and areas of respiratory metaplasia are observed	[72]

HBCs	IOI mouse model	Morphology, proliferation dynamics, immunologic profile, and transcriptomics	The persistent inflammatory state results in a massive removal of neoplastic olfactory epithelial cells and suppression of HBCs differentiation	[73]

HBCs, GBCs	Presbyosmic adult human OE	Single-cell RNA-Seq (scRNA-Seq) analysis	The OE stem cells in presbyosmic patients find inflammation-related changes	[74]

OE (olfactory epithelium)	IOI mice	Histologic analyses. Immunostaining. Electro-olfactogram.	TNF-*α* signaling leads to neuronal dysfunction and axon tract damage leads to loss of ORNs	[75]

IPI3R/NPY	IP3R3tauGFP mice	ELISA quantitative RT-PCR	IP3R3/NPY signaling plays a role in injury-induced OE recovery in aged mice	[76]

CNTF/FAK	CNTF mice	Frozen OE RT-qPCR western blot	FAK inhibition promotes olfactory nerve regeneration via CNTF	[77]

SASP	C57BL/6 mice	qPCR histological analyses	SASP is increased in aging-related olfactory dysfunction	[78]

CYP26B1	OMP-Cyp mice	Histology and immunodetection.	CYP26B1 promotes neurogenesis and inhibits age-related metaplasia	[79]

BDNF	6-month-old male mice	RT-PCR and immunohistochemical technique	BDNF promotes the regeneration of ORNs at different stages of their growth	[80]

Abbreviations: CNTF, ciliary neurotrophic factor; ELISA, enzyme-linked immunosorbent assay; FAK, focal adhesion kinase; IOI, inducible olfactory inflammation; IPI3R, inositol trisphosphate receptor type 3; NPY, neuroproliferative factor neuropeptide Y; OE, olfactory epithelium; RT-PCR, reverse transcriptase polymerase chain reaction; SASP, senescence-associated secretory phenotype.

**Table 4:** Olfactory stem cell-related molecular markers.

Cell type	Marker	Species	References
HBCs	*Δ*Np63	Mouse	[92]
	Lgr5	Human	[127]
	CK5	Mouse	[128]
	CK14	Mouse	[128]
	ICAM1	Mouse	[129]
	PDGFR*α*	Rat	[129]
	Olig2	Rat	[130]

GBCs	SOX2	Mouse	[131]
	PAX6	Mouse	[97]
	Ngn1	Mouse	[112]
	NeuroD1	Rat	[94]
	Ascl	Mouse	[132]

Sustentacular cells and Bowman's glands	CYP2a5	Mouse	[133]
		Human	
	GGT7	Mouse	[133]
		Human	

Bowman's glands	GGT1	Mouse	[133]
		Human	

OS	AN2	Human	[134]
	A2B5	Human	[134]
	Tuj1	Human	[134]
	MAP2	Human	[134]

Mature ORNs	OMP	Mouse	[100]

Immature ORNs	*β*III tubulin	Mouse	[98]

*Note:* CYP2a5, enzymes cytochrome p450–2A6; Lgr5, G-protein-coupled receptor 5; NeuroD1, transcription factor NeuroD1; Olig2, oligodendrocyte transcription factor 2; SOX2, SRY-related HMG-box gene 2; Tuj1, neuron-specific class III beta-tubulin.

Abbreviations: A2B5, A2B5 antigen; AN2, AN2 proteoglycan; CK14, cytokeratin 14; GGT1, g-glutamyltranspeptidase 1; GGT7, g-glutamyltranspeptidase 7; ICAM1, intercellular adhesion molecule 1; MAP2, microtubule-associated protein 2; Ngn1, neurogenin 1; OMP, olfactory marker protein; PAX6, paired box protein 6; PDGFR*α*, platelet-derived growth factor receptor *α*.

## Data Availability

Data sharing is not applicable to this article as no new data were created or analyzed in this study.

## Nomenclature

COVID-19: Coronavirus 2019
PRP: Platelet-rich plasma
CCCRC: Connecticut Chemosensory Clinical Research Center oronasal olfaction test
UPSIT: University of Pennsylvania Smell Identification Test
CRSwNP: Chronic rhinosinusitis with nasal polyps
CRSsNP: Chronic rhinosinusitis without nasal polyps
CT: Computed tomography
HBCs: Horizontal basal cells
GBCs: Global basal cells
ORNs: Olfactory receptor neurons
TGF-B: Transforming growth factor-B
OE: Olfactory epithelium
BDNF: Brain-derived neurotrophic factor
NT-3: Neurotrophic factor-3
GDFII: Growth and differentiation factor II
Lgr5: G-protein coupled receptor 5
Ngn1: Neurogenin 1
Mash 1: Mammalian achaete scute homolog 1
GABA: Gamma-aminobutyric acid
ORN: Olfactory receptor neuron;
IRN: Immature ORN
Hes: Basic helix–loop–helix (bHLH) transcriptional repressors
Pax6: Paired box protein 6
SUS: Sustentacular cells
NeuroD1: Neuronal differentiation 1
*Δ*Np63: Tumor protein p63 with exon replacement at the *Δ*N-terminus
CK5: Cytokeratin 5
OMP: Olfactory marker protein
ONC: Olfactory neuroepithelial cell
TNF-*α*: Tumor necrosis alpha
TNFR1/NF-*κ*ß: Tumor necrosis factor receptor 1/nuclear factor kappa beta
IL-6: Inducing interleukin-6
LRP5/6: Low-density lipoprotein receptor-related protein
GSK3*β*: Glycogen synthase kinase 3 beta
NICD: Notch intracellular domain
*β*-catenin: Beta-catenin
SASP: Senescence-associated secretory phenotype
FAK: Focal adhesion kinase.
